# Morphology of proximal and distal human semitendinosus compartments and the effects of distal tendon harvesting for anterior cruciate ligament reconstruction

**DOI:** 10.1111/joa.13869

**Published:** 2023-04-13

**Authors:** Adam Kositsky, Huub Maas, Rod S. Barrett, Ben Kennedy, Lauri Stenroth, Rami K. Korhonen, Christopher J. Vertullo, Laura E. Diamond, David J. Saxby

**Affiliations:** ^1^ Griffith Centre of Biomedical and Rehabilitation Engineering (GCORE), Menzies Health Institute Queensland Griffith University Gold Coast Queensland Australia; ^2^ Department of Technical Physics University of Eastern Finland Kuopio Finland; ^3^ Department of Human Movement Sciences, Faculty of Behavioural and Movement Sciences, Amsterdam Movement Sciences Vrije Universiteit Amsterdam Amsterdam The Netherlands; ^4^ Mermaid Beach Radiology Gold Coast Queensland Australia; ^5^ Knee Research Australia Gold Coast Queensland Australia

**Keywords:** graft, hamstrings, magnetic resonance imaging, tendinous inscription, tenotomy

## Abstract

The human semitendinosus muscle is characterized by a tendinous inscription separating proximal and distal neuromuscular compartments. As each compartment is innervated by separate nerve branches, potential exists for independent operation and control of compartments. However, the morphology and function of each compartment have not been thoroughly examined in an adult human population. Further, the distal semitendinosus tendon is typically harvested for use in anterior cruciate ligament reconstruction surgery, which induces long‐term morphological changes to the semitendinosus muscle‐tendon unit. It remains unknown if muscle morphological alterations following anterior cruciate ligament reconstruction are uniform between proximal and distal semitendinosus compartments. Here, we performed magnetic resonance imaging on 10 individuals who had undergone anterior cruciate ligament reconstruction involving an ipsilateral distal semitendinosus tendon graft 14 ± 4 months prior, extracting morphological parameters of the whole semitendinosus muscle and each individual compartment from both the (non‐injured) contralateral and surgical legs. In the contralateral leg, volume and length of the proximal compartment were smaller than the distal compartment. No between‐compartment differences in volume or length were found for anterior cruciate ligament reconstructed legs, likely due to greater shortening of the distal compared to the proximal compartment after anterior cruciate ligament reconstruction. The maximal anatomical cross‐sectional area of both compartments was substantially smaller on the anterior cruciate ligament reconstructed leg but did not differ between compartments on either leg. The absolute and relative between‐leg differences in proximal compartment morphology on the anterior cruciate ligament reconstructed leg were strongly correlated with the corresponding between‐leg differences in distal compartment morphological parameters. Specifically, greater between‐leg morphological differences in one compartment were highly correlated with large between‐leg differences in the other compartment, and vice versa for smaller differences. These relationships indicate that despite the heterogeneity in compartment length and volume, compartment atrophy is not independent or random. Further, the tendinous inscription endpoints were generally positioned at the same proximodistal level as the compartment maximal anatomical cross‐sectional areas, providing a wide area over which the tendinous inscription could mechanically interact with compartments. Overall, results suggest the two human semitendinosus compartments are not mechanically independent.

## Graphical abstract

Although being of anatomical intrigue for over 150 years, the human semitendinosus muscle has not been thoroughly examined in a livingadult population. Here, we used magnetic resonance imaging to describe the gross morphology of the two semitendinosus neuromuscular compartments and how each compartment may adapt to distal tendon harvesting for anterior cruciate ligament reconstruction. Despite between‐compartment differences in volume and length in non‐reconstructed legs, we found compartment morphology and adaptations are associated and not random, suggesting the two neuromuscular compartments of the human semitendinosus are not mechanically independent.

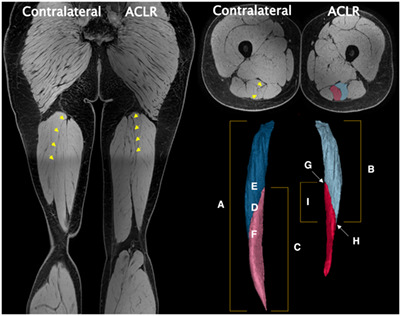

## 1 INTRODUCTION

The structure of the musculus semitendinosus (ST) has been of great intrigue in human and comparative anatomy for over 150 years (Humphry, 1869; Macalister, 1868; Parsons, 1898). The ST of many, but not all (Appleton, 1928), species is characterized by the presence of a tendinous inscription (TI) that separates ST into proximal (ST_prox_) and distal (ST_dist_) neuromuscular compartments, each containing separate nerve innervations (Edgerton et al., 1987; Gans et al., 1989; Hopwood & Butterfield, 1976; Paul, 2001; Roy et al., 1984; Woodley & Mercer, 2005). Although the bicompartmental structure of the human ST has been considered in cadaveric investigations (Barrett, 1962; Garrett et al., 1989; Haberfehlner, Maas, et al., 2016; Kellis et al., 2012; Lee et al., 1988; Markee et al., 1955; van der Made et al., 2015; Wickiewicz et al., 1983; Woodley & Mercer, 2005), in vivo human studies often neglect this unusual design. In vivo studies that have considered both ST compartments have been performed only with typically developing children and those with spasticity (Haberfehlner, Jaspers, et al., 2016; Hanssen et al., 2021), while studies with adult participants only quantified TI location with respect to the ischial tuberosity (Kellis et al., 2012; Kellis & Balidou, 2014). Therefore, information on the bicompartmental morphology of ST in living human adults is currently not available.

Besides its peculiar design, ST has functional and clinical importance. Functionally, ST has been recently suggested to be an essential contributor to upright, bipedal locomotion (Tardieu et al., 2022). Further, sprinters have a relatively large ST (Handsfield et al., 2017), and ST size is correlated with markers of sprint running performance (Takahashi et al., 2021). In orthopedics, the distal ST tendon is routinely harvested as autologous graft tissue, particularly for anterior cruciate ligament reconstruction (ACLR) (Thaunat et al., 2019; Vertullo et al., 2019). Although this surgical procedure essentially sacrifices ST, the ST tendon demonstrates remarkable potential to regenerate and reattach below the knee joint line (Nakamae et al., 2005; Papalia et al., 2015), regaining some level of function. However, after ACLR, the ST muscle belly is substantially shorter, with decreased anatomical cross‐sectional area (ACSA) and volume (Konrath et al., 2016; Makihara et al., 2006; Messer et al., 2020; Morris et al., 2021; Nomura et al., 2015; Snow et al., 2012; Williams et al., 2004). Although the shape of ST, particularly distally, has been qualitatively (Snow et al., 2012) and quantitatively (du Moulin et al., 2023) observed to be different after tendon harvesting for ACLR, it remains unknown if ST_prox_ and ST_dist_ are altered heterogeneously post‐ACLR, particularly as other recent work assessing ST regional ACSA at standardized locations of thigh length (Hjaltadóttir et al., 2022) is confounded by ST shortening post‐ACLR. As ST_prox_ may function primarily at the hip and ST_dist_ predominantly at the knee (Markee et al., 1955), compartment‐specific adaptations, such as shown in musculoskeletal conditions other than ACLR (e.g., children with cerebral palsy; Haberfehlner, Jaspers, et al., 2016), may be relevant for the common and persistent knee flexion weakness following ACLR with an ST graft, even in the presence of ST tendon regeneration (Konrath et al., 2016; Makihara et al., 2006; Nakamae et al., 2005; Nomura et al., 2015; Papalia et al., 2015).

Overall, knowledge of ST compartment morphology in living human adults and potential (in)congruency in between‐compartment adaptions is lacking. Therefore, we used magnetic resonance imaging (MRI) to bilaterally evaluate the morphology of ST, including ST_prox_ and ST_dist_, in adults post‐ACLR with a unilateral distal ST tendon autograft. Specifically, we aimed to assess whole muscle and compartment morphology on the contralateral (non‐surgical) leg, the effects distal ST tendon harvesting has on ST morphology, and how compartments may atrophy with respect to each other, the whole muscle, and the TI. We also aimed to describe the positioning of the TI in relation to compartment and whole muscle morphology and if the positioning may differ on the ACLR leg. We hypothesized the TI would split the ST muscle into two rather homogenous compartments on the non‐ACLR leg. Due to distal ST tendon harvesting, we also hypothesized potential between‐leg morphological differences would be greater in ST_dist_ than ST_prox_.

## 2 METHODS

Ten participants (6 females; age: 27.2 ± 4.9 years; height: 171.6 ± 10.0 cm; mass: 72.6 ± 13.4 kg) were recruited for the study. Participants underwent MRI imaging only after ACLR (424 ± 109 days post‐surgery). Single‐site ultrasound images were also obtained but are reported elsewhere (Kositsky, Barrett, et al., 2022). All participants, of which five had accompanying meniscal lesions, underwent ACLR with a quadrupled ipsilateral ST autograft (see Section 2.1). Exclusion criteria consisted of: ACLR >6 months after initial injury, concomitant harvesting of gracilis tendon for ACLR, previous major knee injuries, neurological disorders, and/or contraindications for MRI scans. Participants were requested to refrain from strenuous exercise commencing 24 h prior to the investigation and provided written informed consent prior to any involvement in the study. The Griffith University Human Research Ethics Committee (2018/839) approved the study, which was carried out in accordance with the Declaration of Helsinki.

### 2.1 Surgical procedures

A fellowship trained orthopedic surgeon (CJV) performed all ACLRs. After application of a tourniquet to the thigh, an anteromedial vertical incision was made over the pes anserinus. The sartorius fascia was then incised to visualize the ST tendon. The tendon was left secured to the distal attachment point and an open‐ended tendon harvester (Linvatec) was used to release the entire distal tendon length from its muscular attachment. Then, the ST tendon was removed from its distal bony attachment with a scalpel. A quadrupled ST graft was formed using a wrapping technique over two Tightrope fixation devices (Arthrex), proximally and distally, and then sutured using Fibrewire (Arthrex) (Vertullo et al., 2019). The femoral tunnel was created via a transportal drilling technique and the tibial tunnel drilled outside‐in. Femoral and tibial fixation with the adjustable fixation devices were undertaken in full extension.

### 2.2 MRI acquisition and data analyses

**FIGURE 1:**
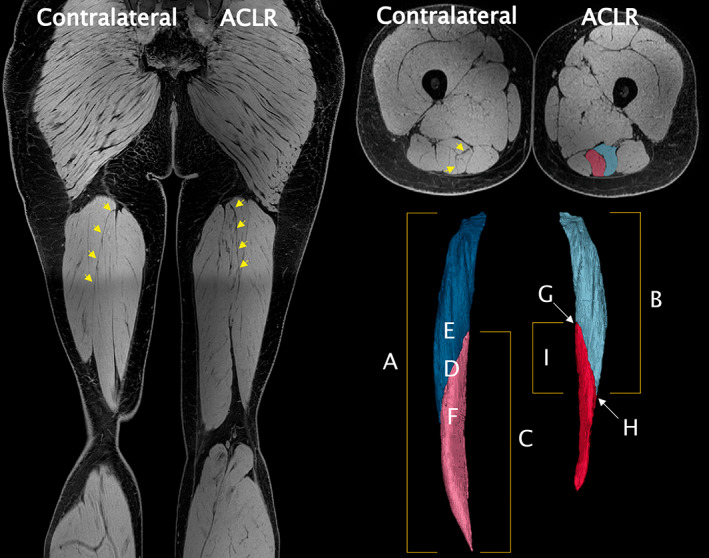
Raw coronal water in‐phase magnetic resonance imaging (MRI) sequence (left). Axially reconstructed MRI image with segmentations of proximal (ST_prox_) and distal (ST_dist_) semitendinosus (ST) compartments overlayed on the anterior cruciate ligament (ACLR) leg (upper right). Example reconstruction of proximal (contralateral: dark blue; ACLR: light blue) and distal (contralateral: pink; ACLR: red) semitendinosus compartments (lower right). Note the full length of the ST muscle is not seen in the coronal slice, reconstructed segmentations are not scaled to the coronal image, and all images are from the same participant, who had ST tendon regeneration and 7.2 cm difference in ST muscle length on the ACLR leg. The tendinous inscription (TI) is indicated with yellow arrows on MRI images. A: ST whole muscle length. B: ST_prox_ compartment length. C: ST_dist_ compartment length. D: ST whole muscle maximal anatomical cross‐sectional area (ACSA_max_). E: ST_prox_ compartment ACSA_max_. F: ST_dist_ compartment ACSA_max_. G: proximal endpoint of TI. H: distal endpoint of TI. I: TI length.

With the participant lying supine, T_1_ Dixon three‐dimensional fast field echo and two‐dimensional proton density magnetic resonance images were acquired with a 3T MRI unit (Ingenia; Phillips). Scan acquisition parameters are summarized in Table 1. For T_1_ Dixon scans, a B1 field map (dual repetition time) was used to minimize signal contrast variation across the field of view. Coronal T_1_ Dixon images were reconstructed into 691 axial slices (1 mm slice thickness) with in‐plane pixel resolution of 0.446 mm using Mimics software (Version 20.0; Materialise). Each compartment was separately traced in approximately every 5 axial slices in the water in‐phase images, with software interpolation used for slices in between. Caution was taken to include as little of the muscle border as possible, and images were manually inspected to ensure interpolation did not cause substantial errors. The ST_prox_ and ST_dist_ masks were also combined and gaps between them filled (i.e., to include the TI, as this is how ST is typically segmented) to create a whole ST mask. Compartment and muscle belly lengths were calculated in the proximodistal axis by multiplying slice thickness (1 mm) by the number of slices in which the respective compartment/muscle was visible (Fukunaga et al., 2001; Messer et al., 2020). The slice containing each compartment's and the entire muscle's largest cross‐sectional value was deemed the compartment/muscle maximal ACSA (ACSA_max_) (Fukunaga et al., 2001; Kositsky et al., 2020). The location of compartment and muscle ACSA_max_ relative to the respective compartment and entire muscle belly length was also determined, with the proximal end of the muscle corresponding to 0% and the distal end to 100%. Compartment and muscle volumes were calculated by multiplying slice thickness (1 mm) by the sum of contiguous ACSAs (Fukunaga et al., 2001; Messer et al., 2020). The position of the proximal (TI_prox_) and distal (TI_dist_) endpoints of the TI was determined relative to the length of each compartment and the entire muscle belly, and the proximodistal length (in the axial imaging plane) of the TI was determined from the number of slices in which ST_prox_ and ST_dist_ overlapped. Examples of all morphological analyses are depicted in Figure 1. The distal ST tendon was considered as regenerated if a tendinous structure was visible on proton density and T_1_ Dixon scans below the knee joint.

**TABLE 1:** Acquisition parameters for magnetic resonance imaging scans.

	T_1_ Dixon	Proton density
Main purpose	Muscle/compartment segmentation	Visualize ST tendon
Acquisition plane	Coronal (3D)	Axial (2D)
Number of stations	Two	One
Number of slices	252	80–95
Slice thickness	1 mm	3 mm
Slice gap	0 mm	0.3 mm
Station overlap	30 mm	—
Repetition time	7.45 ms	2853–3804 ms^a^
Echo time(s)	1.19, 2.37 ms	25 ms
Flip angle	10°	90°
Voxel size	1 × 1 × 1 mm	0.8 × 0.88 mm
Minimum FOV	360 × 450 × 252 mm	200 × 380 mm
Acceleration factor	SENSE 2	SENSE 2.5
Acquisition time	14 min (7 min per station)	5–7 min^a^

Abbreviations: 2D, two‐dimensional; 3D, three‐dimensional; FOV, field of view; ST, semitendinosus.

^a^ Depending on the FOV and the number of slices.

### 2.3 Statistical analyses

Paired samples *t*‐tests were used to assess the between‐leg differences in whole ST muscle morphology (volume, ACSA_max_, length), while the effects of compartment (ST_prox_, ST_dist_) and leg (contralateral, ACLR) on ST compartment volume, ACSA_max_, and length were assessed using full‐factorial, two‐way repeated measures ANOVAs. These statistical analyses were repeated with the regenerated tendon participant subgroup to confirm grouping all participants in a single cohort regardless of tendon regeneration status did not affect our results. The between‐leg differences in TI length and the location of whole ST muscle ACSA_max_ relative to whole muscle and TI lengths were assessed with paired samples *t*‐tests. A two‐way repeated measures ANOVA was performed to assess any within‐ or between‐leg differences in the locations of ST_prox_ ACSA_max_ and TI_prox_ (both relative to muscle length). A separate repeated measures ANOVA was performed comparing the locations of ST_dist_ ACSA_max_ and TI_dist_. For all repeated measures ANOVAs, Bonferroni corrections were applied when significant interactions were found. The between‐leg differences for each compartment morphological parameter (e.g., volume of ST_prox_ on the ACLR leg minus volume of ST_prox_ on the contralateral leg) were determined and Pearson's *r* correlation coefficients used to assess the relationships in between‐leg differences for (i) ST_prox_ and ST_dist_ morphology, (ii) compartment and whole muscle morphology, and (iii) compartment and TI length. All statistical analyses were performed with SPSS (v27, SPSS Inc.). All data are presented as mean ± one standard deviation.

## 3 RESULTS

Whole ST muscle volume (*p* < 0.001), ACSA_max_ (*p* = 0.02), and length (*p* = 0.001) were all smaller on the ACLR compared to the contralateral leg (Table 2). The location of whole muscle ACSA_max_ relative to whole muscle length was more distal on the ACLR leg (contralateral: 40.8 ± 3.3%; ACLR: 49.0 ± 8.4%; *p* = 0.025), but the location of ACSA_max_ along the TI did not differ across legs (contralateral: 36.5 ± 12.0%; ACLR: 39.5 ± 15.8%; *p* = 0.623).

**TABLE 2:** Means and standard deviations of volume, maximal anatomical cross‐sectional area (ACSA_max_), and length of the whole semitendinosus muscle for contralateral and anterior cruciate ligament reconstructed (ACLR) legs. Paired samples *t*‐tests for between‐leg differences were performed for the entire sample and for the regenerated tendon subgroup but not for the non‐regenerated tendon subgroup due to sample size.

	Total (*n* = 10)		Regenerated tendon (*n* = 7)		Non‐regenerated tendon (*n* = 3)	
	Contralateral	ACLR	Contralateral	ACLR	Contralateral	ACLR
Volume	211.9 ± 64.9	138.8 ± 65.7***	234.0 ± 66.2	172.7 ± 44.6^##^	160.3 ± 11.3	59.6 ± 1.5
ACSA_max_	10.8 ± 2.6	9.0 ± 3.3*	11.8 ± 2.6	10.8 ± 2.1	8.6 ± 0.6	5.1 ± 1.1
Length	33.4 ± 4.0	27.2 ± 6.3**	34.0 ± 4.7	30.1 ± 5.0^##^	32.2 ± 0.6	20.5 ± 2.4

*p < 0.05, **p < 0.01, ***p < 0.001 significantly different from the contralateral leg for the entire sample.

^##^ *p* < 0.01 significantly different from the contralateral leg for the regenerated tendon subgroup.

Compartment morphology results are presented in Table 3. A significant interaction (*p* < 0.001) revealed that although volume was smaller for both compartments on the ACLR compared to the contralateral leg (ST_prox_: *p* = 0.003; ST_dist_: *p* < 0.001), ST_dist_ was larger than ST_prox_ on the contralateral (*p* = 0.007), but not the ACLR (*p* = 0.369), leg. There were no differences in ACSA_max_ between compartments on either leg (main effect: *p* = 0.774; interaction: *p* = 0.951). However, a significant main effect (*p* = 0.002) revealed ACSA_max_ to be smaller in both ST compartments on the ACLR compared to the contralateral leg. As with volumetric results, a significant interaction (*p* = 0.002) revealed length to be shorter for both compartments on the ACLR compared to the contralateral leg (ST_prox_: *p* = 0.010; ST_dist_: *p* = 0.001), but ST_dist_ was longer than ST_prox_ on the contralateral (*p* < 0.001), but not the ACLR (*p* = 0.726), leg.

**TABLE 3:** Means and standard deviations of volume, maximal anatomical cross‐sectional area (ACSA_max_), and length of proximal (ST_prox_) and distal (ST_dist_) semitendinosus compartments for contralateral and anterior cruciate ligament reconstructed (ACLR) legs. Repeated measures ANOVAs were performed for the entire sample and for the regenerated tendon subgroup but not for the non‐regenerated tendon subgroup due to sample size. Only between‐compartment statistics are presented in this table. Refer to the main text for main effects and interactions.

	Total (*n* = 10)				Regenerated tendon (*n* = 7)				Non‐regenerated tendon (*n* = 3)			
	Contralateral		ACLR		Contralateral		ACLR		Contralateral		ACLR	
	ST_prox_	ST_dist_	ST_prox_	ST_dist_	ST_prox_	ST_dist_	ST_prox_	ST_dist_	ST_prox_	ST_dist_	ST_prox_	ST_dist_
Volume (cm^3^)	95.1 ± 25.1	116.3 ± 40.5**	65.8 ± 26.2	71.7 ± 40.5	102.0 ± 27.2	131.2 ± 39.9^##^	79.7 ± 16.5	91.5 ± 30.5	78.8 ± 7.4	81.4 ± 4.1	33.3 ± 0.7	25.3 ± 2.1
ACSA_max_ (cm^2^)	9.4 ± 2.0	9.4 ± 2.3	7.1 ± 2.6	7.0 ± 2.9	10.0 ± 2.1	10.3 ± 2.2	8.4 ± 1.9	8.5 ± 2.2	8.1 ± 0.6	7.2 ± 0.2	4.1 ± 0.6	3.8 ± 0.4
Length (cm)	20.3 ± 2.4	23.7 ± 3.0***	18.5 ± 2.2	18.1 ± 5.8	20.5 ± 2.9	24.5 ± 3.3^##^	19.5 ± 1.7	20.9 ± 4.3	19.8 ± 1.1	21.9 ± 0.4	16.1 ± 1.3	11.5 ± 1.9

**p < 0.01, ***p < 0.001 significantly different from ST_prox_ for the entire sample.

^##^ *p* < 0.01 significantly different from ST_prox_ for the regenerated tendon subgroup.

**FIGURE 2:**
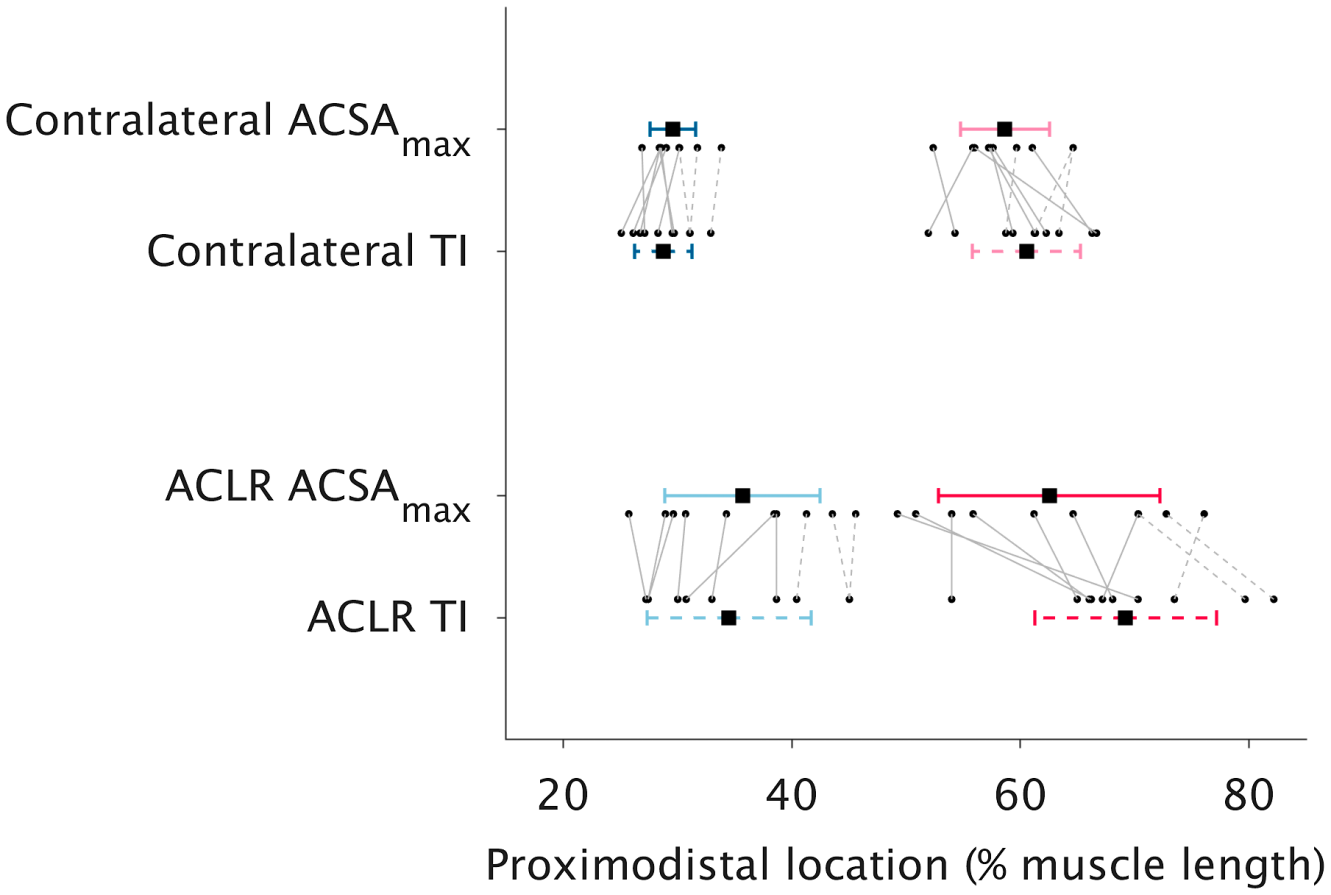
The location of proximal (contralateral: dark blue; anterior cruciate ligament reconstructed: light blue) and distal (contralateral: pink; anterior cruciate ligament reconstructed: red) semitendinosus compartment maximal anatomical cross‐sectional area (ACSA_max_; unbroken lines) compared to tendinous inscription (TI) endpoints (broken lines) for anterior cruciate ligament reconstructed (ACLR) and contralateral legs. Data are presented as means and standard deviations. Dots represent individual data points, with full (regenerated) and broken (non‐regenerated) gray lines connecting data from the same individual.

The proximodistal length of the TI was shorter after ACLR (contralateral: 10.6 ± 2.0 cm; ACLR: 9.3 ± 2.0 cm; *p* = 0.005) but traversed a greater percent of muscle belly length (contralateral: 31.8 ± 5.7%; ACLR: 34.7 ± 4.7%; *p* = 0.015). The TI spanned from 28.8 ± 2.5% (TI_prox_) to 60.5 ± 4.7% (TI_dist_) of muscle belly length on the contralateral leg, and from 34.5 ± 7.2% (TI_prox_) to 69.2 ± 8.0% (TI_dist_) of muscle length on the ACLR leg. This corresponded to 47.6 ± 5.9% of ST_prox_ length to 44.3 ± 7.1% of ST_dist_ length on the contralateral leg and 49.4 ± 6.4% of ST_prox_ length to 53.2 ± 8.7% of ST_dist_ length on the ACLR leg. The location of ST_prox_ ACSA_max_ along whole muscle length (contralateral: 29.6 ± 2.0%; ACLR: 35.7 ± 6.8%) did not differ from TI_prox_ on either leg (main effect: *p* = 0.126; interaction *p* = 0.615), although ST_prox_ ACSA_max_ and TI_prox_ were both more distal on the ACLR leg (main effect: *p* = 0.005) (Figure 2). While the location of ST_dist_ ACSA_max_ with respect to muscle length did not differ between legs (contralateral: 58.6 ± 3.9%; ACLR: 62.5 ± 9.7%; *p* = 0.139), a significant interaction (*p* = 0.021) revealed the location of ST_dist_ ACSA_max_ and TI_dist_ differed only on the ACLR leg (*p* = 0.025), and not the contralateral leg (*p* = 0.211), due to a slightly more distal position (relative to muscle length) of TI_dist_ after ACLR (*p* = 0.007).

**FIGURE 3:**
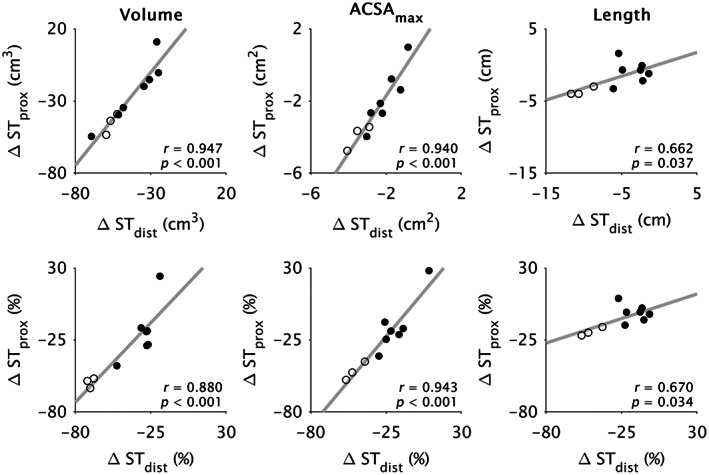
Pearson's correlation coefficients (*r*) for the between‐leg differences in proximal (ST_prox_) versus distal (ST_dist_) semitendinosus compartment volume (left), maximal anatomical cross‐sectional area (ACSA_max_; middle), and length (right), plotted for absolute (upper) and relative (lower) differences. Dots represent individual data points from participants with (filled) and without (unfilled) tendon regeneration. All comparisons were significantly correlated (*p* ≤ 0.037).

**FIGURE 4:**
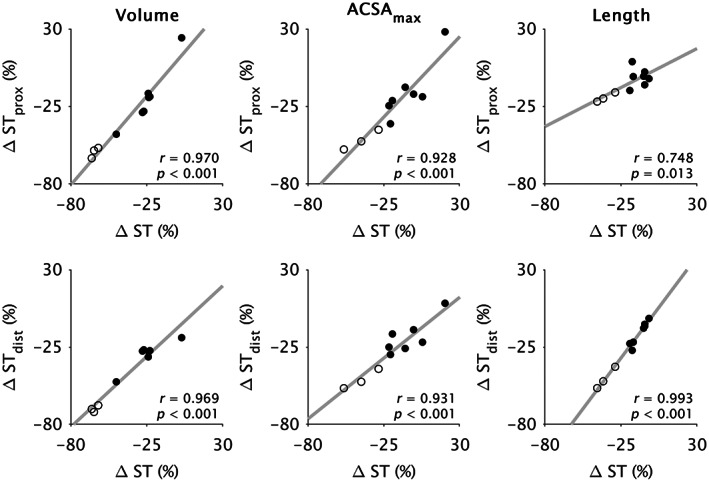
Pearson's correlation coefficients (*r*) for the between‐leg relative differences in whole semitendinosus (ST) muscle versus proximal (ST_prox_; upper) and distal (ST_dist_; lower) ST compartment volume (left), maximal anatomical cross‐sectional area (ACSA_max_; middle), and length (right). Dots represent individual data points from participants with (filled) and without (unfilled) tendon regeneration. All comparisons were significantly correlated (*p* ≤ 0.013).

**FIGURE 5:**
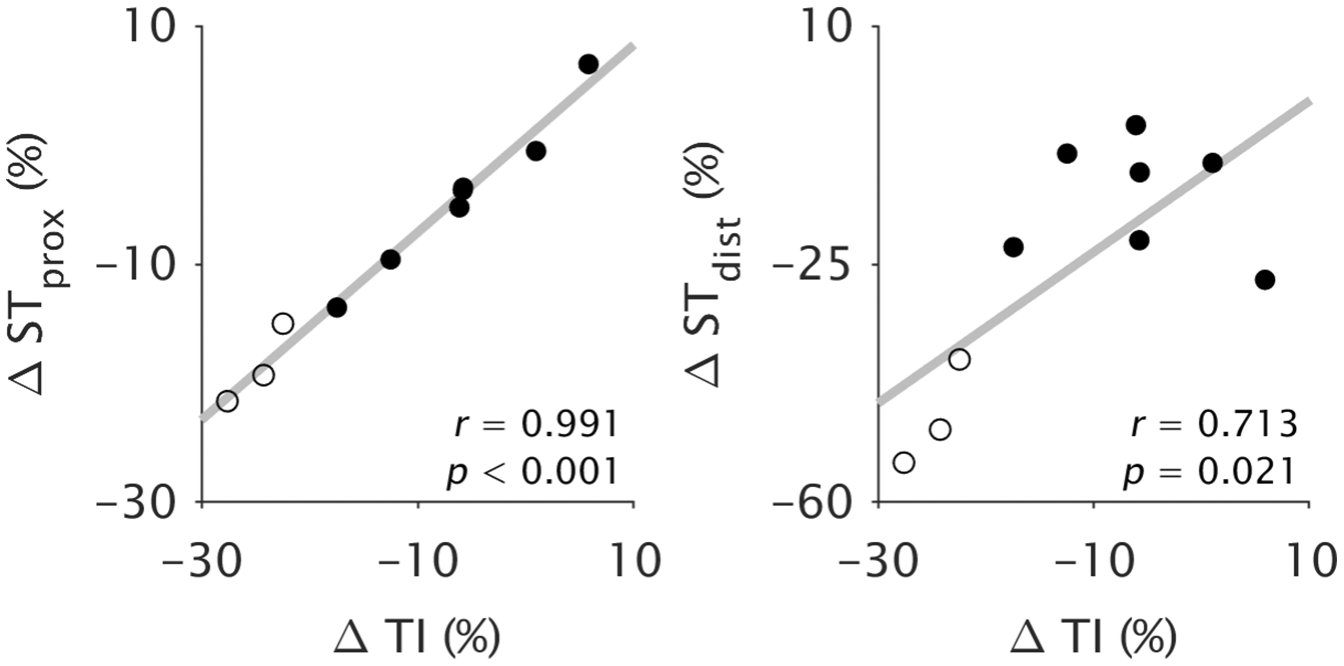
Pearson's correlation coefficients (*r*) for the between‐leg relative differences in tendinous inscription (TI) length versus between‐leg relative differences in proximal (ST_prox_; left) and distal (ST_dist_; right) semitendinosus compartment length. Dots represent individual data points from participants with (filled) and without (unfilled) tendon regeneration. Both correlations were significant (*p* ≤ 0.021).

The between‐leg differences in each morphological parameter (volume, ACSA_max_, length) were highly correlated between compartments (*r* ≥ 0.66; *p* ≤ 0.037; Figure 3). Between‐leg differences in compartment volume and ACSA_max_ were strongly correlated with corresponding whole ST muscle differences (*r* ≥ 0.93; *p* < 0.001; Figure 4), although length differences in ST_dist_ were more strongly correlated with whole ST length differences (*r* = 0.99; *p* < 0.001) than was ST_prox_ (*r* = 0.75; *p* = 0.013). Conversely, the difference in ST_prox_ length was more strongly correlated (*r* = 0.99; *p* < 0.001) with the difference in TI length than was the length difference of ST_dist_ (*r* = 0.71; *p* = 0.021; Figure 5).

Distal ST tendon regeneration was observed in 7 of the 10 participants. In the regenerated tendon subgroup, ST whole muscle volume (*p* = 0.007) and length (*p* = 0.002) differed between legs, although no statistical difference was detected for ACSA_max_ (*p* = 0.185) (Table 2). Compartment morphology results from the regenerated tendon subgroup demonstrated the same main effects and interactions as with the overall sample (Table 3). For volume, a significant interaction (*p* = 0.002) revealed both compartments were smaller on the ACLR compared to the contralateral leg (ST_prox_: *p* = 0.046; ST_dist_: *p* = 0.001) and ST_dist_ was larger than ST_prox_ only on the contralateral leg (contralateral: *p* = 0.004; ACLR: *p* = 0.185). For ACSA_max_, there was a main effect of leg (*p* = 0.031), but not compartment (*p* = 0.724), and no significant interaction (*p* = 0.551). For length, a significant interaction (*p* = 0.030) revealed ST_dist_ was longer than ST_prox_ only on the contralateral leg (contralateral: *p* = 0.002; ACLR: *p* = 0.261), but only ST_dist_ (*p* = 0.003), and not ST_prox_ (*p* = 0.108), was significantly shorter on the ACLR compared to the contralateral leg.

## 4 DISCUSSION

Here, we found ST compartment longitudinal size differs and undergoes compartment‐specific adaptions, whereas compartment maximal radial size does not. More specifically, we found volume and length of ST_dist_ were both larger than ST_prox_ in the contralateral leg, while ACSA_max_ did not differ between compartments. In contrast, although both compartments were substantially smaller in the ACLR compared to the contralateral leg, no between‐compartment differences in morphological parameters were found in the ACLR leg, suggesting larger volume and length changes in ST_dist_ than ST_prox_ following ACLR. We also found the TI endpoints to generally be positioned around the ACSA_max_ of each compartment. These results provide novel insight into the structure and function of the human ST muscle and how ST compartments have potential to be heterogeneously altered, particularly via their overall lengths.

### 4.1 ST morphology on the contralateral leg

Here, the volume of ST_dist_ was greater than ST_prox_ on the contralateral (control) leg (Table 3), whereas previous studies (Haberfehlner, Jaspers, et al., 2016; Haberfehlner, Maas, et al., 2016; Hanssen et al., 2021) have conflicting results with one another regarding between‐compartment volumetric differences, likely due to age and demographic differences between studies (e.g., cadavers, children with or without diseased ST). We also found the proximodistal length of ST_dist_ to be longer than ST_prox_, which is consistent with studies of cats (Bodine et al., 1982; Edgerton et al., 1987; Loeb et al., 1987) and goats (Gans et al., 1989). The fiber and fascicle length of human ST_dist_ was originally reported to be longer than ST_prox_ (Barrett, 1962; Markee et al., 1955), although more recent dissections suggest average fascicle lengths may be equal between compartments (Haberfehlner, Maas, et al., 2016; Kellis et al., 2012; Wickiewicz et al., 1983; Woodley & Mercer, 2005). However, due to the oblique nature of the TI and muscle‐tendon junctions, fascicle length can vary substantially proximodistally (Haberfehlner, Maas, et al., 2016) and depth‐wise (Kellis et al., 2012) within a given compartment. Inferences regarding potential differences in fascicle length from our results are limited as the MRI sequences used only allow for gross morphology to be quantified. Three‐dimensional freehand ultrasound (Haberfehlner, Jaspers, et al., 2016; Haberfehlner, Maas, et al., 2016; Hanssen et al., 2021) and more complex MRI methods, such as diffusion tensor imaging (Bolsterlee et al., 2019), are needed to quantify compartment fascicle lengths in vivo.

Despite being unable to document at the level of fascicles, the between‐compartment differences in length seem to explain the greater volume in ST_dist_ compared to ST_prox_, as ACSA_max_ did not differ between compartments. ACSA_max_, a strong determinant of muscle force, and by extension joint torque (Bamman et al., 2000; Fukunaga et al., 2001), is a good proxy of physiological cross‐sectional area in muscles with little‐to‐no pennation, such as ST (Haberfehlner, Maas, et al., 2016; Makihara et al., 2006). Therefore, despite larger volume in ST_dist_, the lack of a between‐compartment difference in ACSA_max_ suggests the maximal force producing capacity of each compartment does not differ in healthy legs. Further, in accordance with previous reports (Lee et al., 1988; van der Made et al., 2015; Woodley & Mercer, 2005), we found the TI originated at approximately one‐third of muscle length and continued obliquely into the lower half of the ST, although Garrett et al. (1989) found the TI to terminate slightly more proximally. The TI endpoints (TI_prox_, TI_dist_) were centered approximately in the middle of each compartment, connecting regions where compartments are of their maximal size. Considering forces may be transferred between ST compartments (Bodine et al., 1982; Edgerton et al., 1987; Kositsky, Saxby, et al., 2022), the TI is well placed to interact between the two compartments, and the possible functional implications of this placement (e.g., force transmission) are discussed below (see Section 4.3).

Practically, TI endpoints coinciding spatially with compartment ACSA_max_ enables the TI to be used as reference to standardize measures of maximal compartment size, which could also be performed using other, more accessible imaging modalities, such as ultrasonography (Haberfehlner, Maas, et al., 2016; Hanssen et al., 2021; Kositsky et al., 2020). However, given the slightly more proximal position of ST_dist_ ACSA_max_ compared to TI_dist_ in the ACLR leg, assessments of ACSA of ST_dist_ after ACLR should include images proximal to the end of the TI, to ensure ACSA_max_ is obtained. Additionally, as the location of whole ST muscle ACSA_max_ along the TI was highly variable, standardized locations for measures of ST ACSA_max_ (e.g., at 50% of TI length; Haberfehlner, Maas, et al., 2016) should be taken with caution as potential inter‐limb and/or inter‐individual differences at that single location may just be normal variation.

### 4.2 Effects of ACLR on ST morphology

Between‐leg differences in whole ST muscle morphological parameters were comparable with previous studies (Konrath et al., 2016; Makihara et al., 2006; Messer et al., 2020; Nomura et al., 2015; Snow et al., 2012; Williams et al., 2004). Both ST compartments were smaller in volume and shorter in the ACLR leg, but between‐compartment differences in volume and length only for the contralateral, and not ACLR, leg suggests greater volume and length differences in ST_dist_ compared to ST_prox_ on the ACLR leg. Likewise, previous studies have observed greater differences in ST whole muscle volume in individuals with more ST muscle shortening (Nomura et al., 2015; Williams et al., 2004). Greater shortening in ST_dist_ suggests the results of du Moulin et al. (2023), who found greater ACSA_max_ and volume differences in proximal and middle ST regions (based on whole muscle length) post‐ACLR, were influenced by a different contribution of ST_prox_ and ST_dist_ to each muscle region between legs. Despite compartment‐specific adaptations in volume and length and substantial radial atrophy, ACSA_max_ did not differ between compartments in the ACLR leg, implying the maximal force producing capacity was likely reduced by a comparable amount in both ST_prox_ and ST_dist_ after ACLR. The between‐compartment relationships in the between‐leg differences in morphological parameters (Figure 3) confirm compartment morphology and adaptations are not independent, even after such drastic morphological adaptations. However, although the two compartments may be mechanically linked, the more substantial shortening of ST_dist_ may reflect a greater change in the length and/or number of sarcomeres in‐series (Abrams et al., 2000; Crawford, 1977; Van Dyke et al., 2012). According to the force‐length relationship of muscle, muscle fibers in ST_dist_ may thus be too short to produce high levels of force, particularly at highly flexed knee joint angles corresponding to short ST muscle belly lengths (Wickiewicz et al., 1984). Indeed, experimental results demonstrate substantially decreased knee flexion strength in these highly flexed positions post‐ACLR with an ST graft (Makihara et al., 2006; Morris et al., 2021; Nomura et al., 2015). Future studies employing microendoscopy (Pincheira et al., 2022) may be valuable for elucidating compartment‐specific changes at the level of the sarcomere across various joint angles post‐ACLR.

The non‐uniform compartment adaptations post‐ACLR are likely explained by the surgical procedure and the effects of epimuscular myofascial connections (Maas & Sandercock, 2010). The proximal ST tendon and muscle portion remain mechanically connected to surrounding tissues and could still contribute to hip and knee joint torques (de Bruin et al., 2011; Maas & Sandercock, 2008). Thus, ST_prox_ is, particularly in the first months following ACLR, likely to experience greater loading than ST_dist_, which may help maintain its length (Franchi et al., 2022; Wisdom et al., 2015). Further, to harvest the ST tendon, the sartorius fascia is incised and the most distal portion of the ST muscle belly is stripped off the ST tendon with a tendon harvester device. This surgical procedure not only damages the distal muscle end of ST_dist_ but further reduces the myofascial linkages that could maintain some loading through ST_dist_ and physically prevent muscle retraction in the absence of a distal insertion point. Indeed, attenuated strength deficit and less ST muscle shortening were found when the distal ST insertion was maintained by harvesting only partial tendon width (Sasahara et al., 2014). Future work is needed to determine if this partial ST tendon harvesting technique would also mitigate morphological alterations in ST_dist_.

The TI was shorter on the ACLR leg, with a near perfect relationship in the between‐leg differences in ST_prox_ and TI lengths (*r* = 0.991; Figure 5). As ST_prox_ fascicles terminate on the TI and a new set of fascicles (i.e., ST_dist_) originate from the TI (Barrett, 1962; Garrett et al., 1989; Haberfehlner, Maas, et al., 2016; Markee et al., 1955; Woodley & Mercer, 2005), the distoproximal manner of shortening consequent to distal tendon harvest (Street, 1983) suggests ST_dist_ shortening is unlikely to independently have great influence on TI dimensions, as the TI is proximal to the initiation of shortening. In contrast, distoproximal shortening of ST_prox_, whose fascicles are distally attached to the TI, is more likely to be the main regulator of TI shortening. Note that although we only measured its proximodistal length, the overall length of the TI must also be shorter in the ACLR leg due to the concomitant radial muscle atrophy. With a smaller radial size, the overall TI length could be equivalent or greater than the contralateral leg only if the TI was oriented more in‐parallel with the muscle, which would result in an increased proximodistal length and is not what was found in the present study. The shortening of the TI after ACLR may simply be slackening or crimping as a consequence of geometric constraints or may be plastic modulations, as demonstrated in other aponeuroses after (un)loading conditions (Lee et al., 2006; Wakahara et al., 2015), and should be examined in more detail in future investigations.

Between‐leg differences in compartment morphology were still evident in the regenerated tendon subgroup (Table 3), and between‐compartment relationships held even when stratified by tendon regeneration status (Figure 3). However, whole ST muscle ACSA_max_ did not statistically differ between legs in the regenerated tendon subgroup, which may stem from the large within‐sample variation (between‐leg mean difference: −7.3 ± 14.9%), but may be a result previously overlooked as studies assessing ST ACSA_max_ post‐ACLR did not statistically test this parameter in a regenerated participant subgroup against their contralateral or control legs (du Moulin et al., 2023; Konrath et al., 2016; Snow et al., 2012; Williams et al., 2004) or used an average of five 3.6 mm slices when determining ACSA_max_ (Messer et al., 2020). Although unable to statistically compare between subgroups, non‐regenerated tendon individuals (*n* = 3) seemed to have greater between‐leg and between‐compartment morphological differences (Figures 3, 4, 5; Tables 2 and 3), which is consistent with previous reports of greater shortening and muscle atrophy after a lack of tendon regeneration (Crawford, 1977; Davenport & Ranson, 1930; du Moulin et al., 2023; Konrath et al., 2016; Nomura et al., 2015). While the material and compositional properties possibly differ between regenerated and native tendons (Papalia et al., 2015), the more substantial morphological changes when the ST tendon does not regenerate highlights the clinical and functional importance of facilitating tendon regrowth after full tendon harvesting, as re‐establishing a distal insertion point provides a functional mechanical linkage between the muscle, its surroundings, and bone, allowing greater loading and attenuation of ST atrophy. Studies targeting improved ST tendon healing (e.g., du Moulin et al., 2022) may thus also be beneficial for maintaining ST whole muscle and compartment morphology.

### 4.3 Role and function of the TI

The placement of an oblique, full‐thickness TI within the human (and a variety of mammalian) ST has been puzzling anatomists for over a century (Humphry, 1869; Parsons, 1898). Ontogenetically, it is thought the TI marks the fusion between two separately developing anlagen (Bardeen, 1906; Macalister, 1868) and is possibly a neomorph (Appleton, 1928) resulting from the crossing of two muscles (Haines, 1934). However, Parsons (1898) noted a TI is not always present at the union of two muscle heads and thus there may be further morphogenetic explanations. Parsons' sentiments were supported by later works finding a small number of fascicles bridge the TI and course from ST_prox_ to distal tendon insertion (Loeb et al., 1987; Markee et al., 1955; Woodley & Mercer, 2005), and that a TI separating compartments can also be present even when ST_prox_ is itself divided into two (dorsal and ventral) heads (Roy et al., 1984). As fascicles generally terminate (ST_prox_) or originate (ST_dist_) on the TI and to date intrafascicularly terminating muscle fibers within the human ST have not been observed (Barrett, 1962; Woodley & Mercer, 2005), the TI potentially serves to simply connect in‐series muscle fibers (Humphry, 1872; Trotter et al., 1995). Connecting serial muscle fibers through a TI would allow fibers to be of various lengths and experience varying levels of strain (Loeb et al., 1987), potentially reducing the risk of fiber damage without severely affecting ST function, given its wide joint‐level operating range (Cutts, 1989; Peters & Rick, 1977), and allow for deep‐to‐superficial subunits within a given compartment (Bodine et al., 1982; Chanaud et al., 1991; Kellis et al., 2012). However, intrafascicularly terminating muscle fibers have been found in other human muscles with TIs (e.g., rectus abdominis; Cullen & Brödel, 1937; Woodley et al., 2007) and within ST compartments in other mammals whose ST contains a TI (Gans et al., 1989; Loeb et al., 1987). Therefore, even if human ST fibers do span entire fascicles, the TI seems to have another, main functional role than to just connect serial fibers.

Using shear‐wave elastography, we recently indirectly demonstrated passive forces do not differ between human ST compartments, although it was unclear if forces were independently but equally developed or transmitted from one compartment to the other, resulting in equilibrium across the whole muscle (Kositsky, Saxby, et al., 2022). These findings corroborate data from cat ST, whereby forces appear to be transferred between compartments (Bodine et al., 1982; Edgerton et al., 1987). Here, we document the TI is advantageously positioned to possibly assist in force transmission by connecting the largest regions of each compartment (Figure 2), and this placement generally remains after the substantial gross morphological changes induced by harvesting the ST tendon for ACLR. As efficient force transmission from muscle fibers to the connective tissue network occurs through shear at fiber ends (Purslow, 2020), the oblique arrangement of the TI provides a geometrical design facilitating shearing at the junction between fiber and connective tissue that would not be possible if the TI was completely transverse or coursing in the fascicle direction. The consequence of such an anatomical arrangement could allow for re‐distribution and transmission of forces across fascicles of each compartment, as suggested by Kellis et al. (2012). In support of a force transmission role of the TI, muscle fiber‐TI connections have been reported to be comparable with myotendinous junctions (Hijikata & Ishikawa, 1997), and the TI of other muscles, such as in the cat neck, has been shown to house and/or be surrounded by Golgi tendon organs and muscle spindles (Richmond & Abrahams, 1975a, 1975b). Should the TI of ST also contain these sensory receptors, detection of local forces by Golgi tendon organs (Maas et al., 2022) and muscle spindles (Smilde et al., 2016) combined with the potential for asynchronous activation (English & Weeks, 1987; Hutchison et al., 1989) and unequal strains (Edgerton et al., 1987; Markee et al., 1955) between compartments provides a mechanism by which the central nervous system could use the TI to regulate compartmental force and stiffness to control intercompartmental coordination and enable efficient force transmission between compartments. Future studies combining complex computational models assessing force transmission (Sharafi & Blemker, 2011; Zhang & Gao, 2012) and muscle fiber interaction with internal aponeuroses (Knaus et al., 2022) may be able to clarify the main functional role(s) of the TI.

### 4.4 Limitations

We only used the contralateral, non‐surgical leg as the control measure. However, unlike the quadriceps, hamstring morphology on the injured leg remains unchanged following anterior cruciate ligament injury alone (Kariya et al., 1989; Konishi et al., 2012; Lorentzon et al., 1989; Williams et al., 2004), and after ACLR the morphology of ST on the non‐injured leg does not differ compared to pre‐surgical (Williams et al., 2004) and control (du Moulin et al., 2023; Morris et al., 2021) groups. Additionally, the substantial between‐leg differences in morphology found here compare well with previous literature (Konrath et al., 2016; Makihara et al., 2006; Messer et al., 2020; Nomura et al., 2015; Williams et al., 2004) and exceed bilateral asymmetry measures previously reported for ST (Kulas et al., 2018; Speedtsberg et al., 2022; Williams et al., 2004). Therefore, using the contralateral leg as the baseline control in a sample of 10 participants was unlikely to have influenced the results. Further, compartment length was quantified by the proximodistal length of the respective compartment. As the TI is a complex three‐dimensional structure, compartments are comprised of fascicles of various lengths (Haberfehlner, Maas, et al., 2016; Kellis et al., 2012) and thus compartment length may not accurately represent fiber or fascicle length. Therefore, we do not make any concrete conclusions at length scales below proximodistal compartment length as they were not possible to assess from our MRI scans. Finally, the ACLR surgical intervention induces secondary trauma at the knee joint and is thus more complex than regular tenotomy. However, slightly greater changes in ACSA are seen in the distal compared to proximal gracilis muscle when its distal tendon is harvested for shoulder reconstruction (Flies et al., 2020). Therefore, the results found in the present study are likely due to the ST tendon harvest for the ACLR procedure rather than post‐ACLR immobilization and disuse, but should be confirmed in future studies assessing ST compartment alterations after an ST tendon autograft has been used for reconstructing other lower (Cody et al., 2018; Stenroos & Brinck, 2020) and upper (Ranne et al., 2020; Virtanen et al., 2014) limb tendons. The compartment alterations in the ACLR leg may also not be representative of other (un)loading conditions, whose adaptations can also be assessed using the MRI acquisition parameters presented in this study.

## 5 CONCLUSIONS

The proximal and distal compartments of the human ST muscle appear to be modified in a non‐uniform manner following harvest for ACLR. However, the heterogenous changes in length do not affect the homogeneity in compartment maximal radial size. The location of the TI with respect to compartment morphology provides a wide area over which this connective tissue sheath could mediate the mechanical interaction of ST compartments. Overall, these results suggest the proximal and distal compartments of the human ST muscle are not mechanically independent.

## AUTHOR CONTRIBUTIONS

Adam Kositsky, Huub Maas, Laura E. Diamond, and David J. Saxby conceptualized and designed the study. Christopher J. Vertullo performed the surgical intervention. Adam Kositsky and Ben Kennedy collected the data. Adam Kositsky analyzed the data. Adam Kositsky, Huub Maas, Rod S. Barrett, Lauri Stenroth, and David J. Saxby interpreted the data. Lauri Stenroth, Rami K. Korhonen, Laura E. Diamond, and David J. Saxby provided supervision. Adam Kositsky drafted the manuscript and created the figures. All authors critically revised and approved the final manuscript.

## CONFLICT OF INTEREST STATEMENT

The authors have no conflict of interest to declare.

## Data Availability

The data that support the findings of this study are available from the corresponding author upon reasonable request.

## References

[joa13869-bib-0001] Abrams, R.A. , Tsai, A.M. , Watson, B. , Jamali, A. & Lieber, R.L. (2000) Skeletal muscle recovery after tenotomy and 7‐day delayed muscle length restoration. Muscle & Nerve, 23(5), 707–714. Available from: 10.1002/(SICI)1097-4598(200005)23:5<707::AID-MUS7>3.0.CO;2-T 10797393

[joa13869-bib-0002] Appleton, A.B. (1928) The muscles and nerves of the post‐axial region of the tetrapod thigh: part II. Journal of Anatomy, 62(Pt 4), 401–438. 17104202 PMC1249987

[joa13869-bib-0003] Bamman, M.M. , Newcomer, B.R. , Larson‐Meyer, D.E. , Weinsier, R.L. & Hunter, G.R. (2000) Evaluation of the strength‐size relationship in vivo using various muscle size indices. Medicine & Science in Sports & Exercise, 32(7), 1307–1313. Available from: 10.1097/00005768-200007000-00019 10912898

[joa13869-bib-0004] Bardeen, C.R. (1906) Development and variation of the nerves and the musculature of the inferior extremity and of the neighboring regions of the trunk in man. American Journal of Anatomy, 6(1), 259–390. Available from: 10.1002/aja.1000060108

[joa13869-bib-0005] Barrett, B. (1962) The length and mode of termination of individual muscle fibres in the human sartorius and posterior femoral muscles. Acta Anatomica, 48, 242–257. Available from: 10.1159/000141843 13865240

[joa13869-bib-0006] Bodine, S.C. , Roy, R.R. , Meadows, D.A. , Zernicke, R.F. , Sacks, R.D. , Fournier, M. et al. (1982) Architectural, histochemical, and contractile characteristics of a unique biarticular muscle: the cat semitendinosus. Journal of Neurophysiology, 48(1), 192–201. Available from: 10.1152/jn.1982.48.1.192 7119845

[joa13869-bib-0007] Bolsterlee, B. , D'Souza, A. & Herbert, R.D. (2019) Reliability and robustness of muscle architecture measurements obtained using diffusion tensor imaging with anatomically constrained tractography. Journal of Biomechanics, 86, 71–78. Available from: 10.1016/j.jbiomech.2019.01.043 30739766

[joa13869-bib-0008] Chanaud, C.M. , Pratt, C.A. & Loeb, G.E. (1991) Functionally complex muscles of the cat hindlimb. V. the roles of histochemical fiber‐type regionalization and mechanical heterogeneity in differential muscle activation. Experimental Brain Research, 85(2), 300–313. Available from: 10.1007/BF00229408 1832646

[joa13869-bib-0009] Cody, E.A. , Karnovsky, S.C. , DeSandis, B. , Tychanski Papson, A. , Deland, J.T. & Drakos, M.C. (2018) Hamstring autograft for foot and ankle applications. Foot & Ankle International, 39(2), 189–195. Available from: 10.1177/1071100717738220 29171284

[joa13869-bib-0010] Crawford, G.N.C. (1977) Some effects of tenotomy on adult striated muscles. Journal of Anatomy, 123(2), 389–396. 870475 PMC1234538

[joa13869-bib-0011] Cullen, T.S. & Brödel, M. (1937) Lesions of the rectus abdominis muscle simulating an acute intra‐abdominal condition. I. Anatomy of the rectus muscle. Bulletin of the Johns Hopkins Hospital, 61, 295–316.

[joa13869-bib-0012] Cutts, A. (1989) Sarcomere length changes in muscles of the human thigh during walking. Journal of Anatomy, 166, 77–84. 2621149 PMC1256741

[joa13869-bib-0013] Davenport, H.K. & Ranson, S.W. (1930) Contracture resulting from tenotomy. Archives of Surgery, 21(6), 995–1014. Available from: 10.1001/archsurg.1930.01150180111007

[joa13869-bib-0014] de Bruin, M. , Smeulders, M.J.C. & Kreulen, M. (2011) Flexor carpi ulnaris tenotomy alone does not eliminate its contribution to wrist torque. Clinical Biomechanics, 26(7), 725–728. Available from: 10.1016/j.clinbiomech.2011.03.007 21470727

[joa13869-bib-0015] du Moulin, W. , Bourne, M. , Diamond, L.E. , Konrath, J. , Vertullo, C. , Lloyd, D. et al. (2023) Shape differences in the semitendinosus following tendon harvesting for anterior cruciate ligament reconstruction. Journal of Orthopaedic Research, 41(1), 44–53. Available from: 10.1002/jor.25337 35434842 PMC10084140

[joa13869-bib-0016] du Moulin, W. , Kositsky, A. , Bourne, M.N. , Diamond, L.E. , Tudor, F. , Vertullo, C. et al. (2022) Study protocol for double‐blind, randomised placebo‐controlled trial evaluating semitendinosus function and morbidity following tendon harvesting for anterior cruciate ligament reconstruction augmented by platelet‐rich plasma. BMJ Open, 12(9), e061701. Available from: 10.1136/bmjopen-2022-061701 PMC9486297 36123079

[joa13869-bib-0017] Edgerton, V.R. , Bodine, S.C. & Roy, R.R. (1987) Muscle architecture and performance: stress and strain relationships in a muscle with two compartments arranged in series. In: Marconnet, P. & Komi, P.V. (Eds.) Medicine and sport science: muscular function in exercise and training. Basel: Karger, pp. 12–23.

[joa13869-bib-0018] English, A.W. & Weeks, O.I. (1987) An anatomical and functional analysis of cat biceps femoris and semitendinosus muscles. Journal of Morphology, 191(2), 161–175. Available from: 10.1002/jmor.1051910207 3560234

[joa13869-bib-0019] Flies, A. , Denecke, T. , Kraus, N. , Kruppa, P. , Provencher, M.T. , Becker, R. et al. (2020) Tendon regeneration and muscle hypotrophy after isolated Gracilis tendon harvesting—a pilot study. Journal of Experimental Orthopaedics, 7, 19. Available from: 10.1186/s40634-020-00236-8 32266508 PMC7138873

[joa13869-bib-0020] Franchi, M.V. , Sarto, F. , Simunič, B. , Pišot, R. & Narici, M.V. (2022) Early changes of hamstrings morphology and contractile properties during 10 d of complete inactivity. Medicine & Science in Sports & Exercise, 54(8), 1346–1354. Available from: 10.1249/MSS.0000000000002922 35324511

[joa13869-bib-0021] Fukunaga, T. , Miyatani, M. , Tachi, M. , Kouzaki, M. , Kawakami, Y. & Kanehisa, H. (2001) Muscle volume is a major determinant of joint torque in humans. Acta Physiologica Scandinavica, 172(4), 249–255. Available from: 10.1046/j.1365-201x.2001.00867.x 11531646

[joa13869-bib-0022] Gans, C. , Loeb, G.E. & de Vree, F. (1989) Architecture and consequent physiological properties of the semitendinosus muscle in domestic goats. Journal of Morphology, 199(3), 287–297. Available from: 10.1002/jmor.1051990305 2709419

[joa13869-bib-0023] Garrett, W.E. , Rich, F.R. , Nikolaou, P.K. & Vogler, J.B. (1989) Computed tomography of hamstring muscle strains. Medicine & Science in Sports & Exercise, 21(5), 506–514. 2607944

[joa13869-bib-0024] Haberfehlner, H. , Jaspers, R.T. , Rutz, E. , Becher, J.G. , Harlaar, J. , van der Sluijs, J.A. et al. (2016) Knee moment‐angle characteristics and semitendinosus muscle morphology in children with spastic paresis selected for medial hamstring lengthening. PLoS One, 11(11), e0166401. Available from: 10.1371/journal.pone.0166401 27861523 PMC5115739

[joa13869-bib-0025] Haberfehlner, H. , Maas, H. , Harlaar, J. , Becher, J.G. , Buizer, A.I. & Jaspers, R.T. (2016) Freehand three‐dimensional ultrasound to assess semitendinosus muscle morphology. Journal of Anatomy, 229(4), 591–599. Available from: 10.1111/joa.12501 27271461 PMC5013067

[joa13869-bib-0026] Haines, R.W. (1934) The homologies of the flexor and adductor muscles of the thigh. Journal of Morphology, 56(1), 21–49. Available from: 10.1002/jmor.1050560103

[joa13869-bib-0027] Handsfield, G.G. , Knaus, K.R. , Fiorentino, N.M. , Meyer, C.H. , Hart, J.M. & Blemker, S.S. (2017) Adding muscle where you need it: non‐uniform hypertrophy patterns in elite sprinters. Scandinavian Journal of Medicine and Science in Sports, 27(10), 1050–1060. Available from: 10.1111/sms.12723 27373796

[joa13869-bib-0028] Hanssen, B. , De Beukelaer, N. , Schless, S.‐H. , Cenni, F. , Bar‐On, L. , Peeters, N. et al. (2021) Reliability of processing 3‐D freehand ultrasound data to define muscle volume and Echo‐intensity in pediatric lower limb muscles with typical development or with spasticity. Ultrasound in Medicine and Biology, 47(9), 2702–2712. Available from: 10.1016/j.ultrasmedbio.2021.04.028 34112554

[joa13869-bib-0029] Hijikata, T. & Ishikawa, H. (1997) Functional morphology of serially linked skeletal muscle fibers. Acta Anatomica, 159(2–3), 99–107. Available from: 10.1159/000147972 9575360

[joa13869-bib-0030] Hjaltadóttir, A.Þ. , Hafsteinsson, D. , Árnason, Á. & Briem, K. (2022) Musculoskeletal ultrasound imaging of proximal and distal hamstrings cross sectional area in individuals with history of anterior cruciate ligament reconstruction. Physiotherapy Theory and Practice, 1–7. Available from: 10.1080/09593985.2022.2135980 36263941

[joa13869-bib-0031] Hopwood, P.R. & Butterfield, R.M. (1976) The musculature of the proximal pelvic limb of the Eastern Grey Kangaroo Macropus major (Shaw) Macropus giganteus (Zimm). Journal of Anatomy, 121(2), 259–277. 931780 PMC1231798

[joa13869-bib-0032] Humphry, G.M. (1869) On the disposition and homologies of the extensor and flexor muscles of the leg and forearm. Journal of Anatomy and Physiology, 3(Pt 2), 320–334. PMC1318685 17230808

[joa13869-bib-0033] Humphry, G.M. (1872) Lectures on human myology. Lecture III. June 21st, 1872. British Medical Journal, 2(603), 57–60. Available from: 10.1136/bmj.2.603.57 PMC2296936 20746701

[joa13869-bib-0034] Hutchison, D.L. , Roy, R.R. , Bodine‐Fowler, S. , Hodgson, J.A. & Edgerton, V.R. (1989) Electromyographic (EMG) amplitude patterns in the proximal and distal compartments of the cat semitendinosus during various motor tasks. Brain Research, 479(1), 56–64. Available from: 10.1016/0006-8993(89)91335-8 2924153

[joa13869-bib-0035] Kariya, Y. , Itoh, M. , Nakamura, T. , Yagi, K. & Kurosawa, H. (1989) Magnetic resonance imaging and spectroscopy of thigh muscles in cruciate ligament insufficiency. Acta Orthopaedica Scandinavica, 60(3), 322–325. Available from: 10.3109/17453678909149286 2750508

[joa13869-bib-0036] Kellis, E. & Balidou, A. (2014) In vivo examination of the morphology of the tendinous inscription of the human semitendinosus muscle: gender and joint position effects. Journal of Morphology, 275(1), 57–64. Available from: 10.1002/jmor.20196 24127198

[joa13869-bib-0037] Kellis, E. , Galanis, N. , Natsis, K. & Kapetanos, G. (2012) In vivo and in vitro examination of the tendinous inscription of the human semitendinosus muscle. Cells, Tissues, Organs, 195(4), 365–376. Available from: 10.1159/000327574 21828998

[joa13869-bib-0038] Knaus, K.R. , Handsfield, G.G. & Blemker, S.S. (2022) A 3D model of the soleus reveals effects of aponeuroses morphology and material properties on complex muscle fascicle behavior. Journal of Biomechanics, 130, 110877. Available from: 10.1016/j.jbiomech.2021.110877 34896789 PMC8841064

[joa13869-bib-0039] Konishi, Y. , Kinugasa, R. , Oda, T. , Tsukazaki, S. & Fukubayashi, T. (2012) Relationship between muscle volume and muscle torque of the hamstrings after anterior cruciate ligament lesion. Knee Surgery, Sports Traumatology, Arthroscopy, 20(11), 2270–2274. Available from: 10.1007/s00167-012-1888-7 22258654

[joa13869-bib-0040] Konrath, J.M. , Vertullo, C.J. , Kennedy, B.A. , Bush, H.S. , Barrett, R.S. & Lloyd, D.G. (2016) Morphologic characteristics and strength of the hamstring muscles remain altered at 2 years after use of a hamstring tendon graft in anterior cruciate ligament reconstruction. American Journal of Sports Medicine, 44(10), 2589–2598. Available from: 10.1177/0363546516651441 27432052

[joa13869-bib-0041] Kositsky, A. , Barrett, R.S. , du Moulin, W. , Diamond, L.E. & Saxby, D.J. (2022) Semitendinosus muscle morphology in relation to surface electrode placement in anterior cruciate ligament reconstructed and contralateral legs. Frontiers in Sports and Active Living, 4, 959966. Available from: 10.3389/fspor.2022.959966 36425302 PMC9680646

[joa13869-bib-0042] Kositsky, A. , Gonçalves, B.A.M. , Stenroth, L. , Barrett, R.S. , Diamond, L.E. & Saxby, D.J. (2020) Reliability and validity of ultrasonography for measurement of hamstring muscle and tendon cross‐sectional area. Ultrasound in Medicine and Biology, 46(1), 55–63. Available from: 10.1016/j.ultrasmedbio.2019.09.013 31668942

[joa13869-bib-0043] Kositsky, A. , Saxby, D.J. , Lesch, K.J. , Barrett, R.S. , Kröger, H. , Lahtinen, O. et al. (2022) In vivo assessment of the passive stretching response of the bicompartmental human semitendinosus muscle using shear‐wave elastography. Journal of Applied Physiology, 132(2), 438–447. Available from: 10.1152/japplphysiol.00473.2021 34941438 PMC8799393

[joa13869-bib-0044] Kulas, A.S. , Schmitz, R.J. , Shultz, S.J. , Waxman, J.P. , Wang, H.M. , Kraft, R.A. et al. (2018) Bilateral quadriceps and hamstrings muscle volume asymmetries in healthy individuals. Journal of Orthopaedic Research, 36(3), 963–970. Available from: 10.1002/jor.23664 28755488

[joa13869-bib-0045] Lee, H.‐D. , Finni, T. , Hodgson, J.A. , Lai, A.M. , Edgerton, V.R. & Sinha, S. (2006) Soleus aponeurosis strain distribution following chronic unloading in humans: an in vivo MR phase‐contrast study. Journal of Applied Physiology, 100(6), 2004–2011. Available from: 10.1152/japplphysiol.01085.2005 16424072

[joa13869-bib-0046] Lee, T.C. , O'Driscoll, K.J. , McGettigan, P. , Moraes, D. , Ramphall, S. & O'Brien, M. (1988) The site of the tendinous interruption in semitendinosus in man. Journal of Anatomy, 157, 229–231. 3198479 PMC1261957

[joa13869-bib-0047] Loeb, G.E. , Pratt, C.A. , Chanaud, C.M. & Richmond, F.J.R. (1987) Distribution and innervation of short, interdigitated muscle fibers in parallel‐fibered muscles of the cat hindlimb. Journal of Morphology, 191(1), 1–15. Available from: 10.1002/jmor.1051910102 3820310

[joa13869-bib-0048] Lorentzon, R. , Elmqvist, L.G. , Sjöström, M. , Fagerlund, M. & Fuglmeyer, A.R. (1989) Thigh musculature in relation to chronic anterior cruciate ligament tear: muscle size, morphology, and mechanical output before reconstruction. American Journal of Sports Medicine, 17(3), 423–429. Available from: 10.1177/036354658901700318 2729494

[joa13869-bib-0049] Maas, H. , Noort, W. , Smilde, H.A. , Vincent, J.A. , Nardelli, P. & Cope, T.C. (2022) Detection of epimuscular myofascial forces by Golgi tendon organs. Experimental Brain Research, 240(1), 147–158. Available from: 10.1007/s00221-021-06242-1 34677632 PMC8803698

[joa13869-bib-0050] Maas, H. & Sandercock, T.G. (2008) Are skeletal muscles independent actuators? Force transmission from soleus muscle in the cat. Journal of Applied Physiology, 104(6), 1557–1567. Available from: 10.1152/japplphysiol.01208.2007 18339889

[joa13869-bib-0051] Maas, H. & Sandercock, T.G. (2010) Force transmission between synergistic skeletal muscles through connective tissue linkages. Journal of Biomedicine and Biotechnology, 2010, 575672. Available from: 10.1155/2010/575672 20396618 PMC2853902

[joa13869-bib-0052] Macalister, A. (1868) On the homologies of the flexor muscles of the vertebrate limb. Journal of Anatomy and Physiology, 2(2), 283–289. PMC1318615 17230766

[joa13869-bib-0053] Makihara, Y. , Nishino, A. , Fukubayashi, T. & Kanamori, A. (2006) Decrease of knee flexion torque in patients with ACL reconstruction: combined analysis of the architecture and function of the knee flexor muscles. Knee Surgery, Sports Traumatology, Arthroscopy, 14(4), 310–317. Available from: 10.1007/s00167-005-0701-2 16208458

[joa13869-bib-0054] Markee, J.E. , Logue, J.T. , Williams, M. , Stanton, W.B. , Wrenn, R.N. & Walker, L.B. (1955) Two‐joint muscles of the thigh. Journal of Bone & Joint Surgery, 37(1), 125–142. Available from: 10.2106/00004623-195537010-00015 13233278

[joa13869-bib-0055] Messer, D.J. , Shield, A.J. , Williams, M.D. , Timmins, R.G. & Bourne, M.N. (2020) Hamstring muscle activation and morphology are significantly altered 1‐6 years after anterior cruciate ligament reconstruction with semitendinosus graft. Knee Surgery, Sports Traumatology, Arthroscopy, 28(3), 733–741. Available from: 10.1007/s00167-019-05374-w 31030253

[joa13869-bib-0056] Morris, N. , Jordan, M.J. , Sumar, S. , Adrichem, B. , Heard, M. & Herzog, W. (2021) Joint angle‐specific impairments in rate of force development, strength, and muscle morphology after hamstring autograft. Translational Sports Medicine, 4(1), 104–114. Available from: 10.1002/tsm2.189

[joa13869-bib-0057] Nakamae, A. , Deie, M. , Yasumoto, M. , Adachi, N. , Kobayashi, K. , Yasunaga, Y. et al. (2005) Three‐dimensional computed tomography imaging evidence of regeneration of the semitendinosus tendon harvested for anterior cruciate ligament reconstruction: a comparison with hamstring muscle strength. Journal of Computer Assisted Tomography, 29(2), 241–245. Available from: 10.1097/01.rct.0000153779.86663.92 15772546

[joa13869-bib-0058] Nomura, Y. , Kuramochi, R. & Fukubayashi, T. (2015) Evaluation of hamstring muscle strength and morphology after anterior cruciate ligament reconstruction. Scandinavian Journal of Medicine & Science in Sports, 25(3), 301–307. Available from: 10.1111/sms.12205 24646218

[joa13869-bib-0059] Papalia, R. , Franceschi, F. , D'Adamio, S. , Diaz Balzani, L. , Maffulli, N. & Denaro, V. (2015) Hamstring tendon regeneration after harvest for anterior cruciate ligament reconstruction: a systematic review. Arthroscopy, 31(6), 1169–1183. Available from: 10.1016/j.arthro.2014.11.015 25557918

[joa13869-bib-0060] Parsons, F.G. (1898) The muscles of mammals, with special relation to human myology: a course of lectures delivered at the Royal College of Surgeons of England. Lecture II. The muscles of the shoulder and fore‐limb. Journal of Anatomy and Physiology, 32(Pt 4), 721–752. PMC1327923 17232340

[joa13869-bib-0061] Paul, A.C. (2001) Muscle length affects the architecture and pattern of innervation differently in leg muscles of mouse, Guinea pig, and rabbit compared to those of human and monkey muscles. Anatomical Record, 262(3), 301–309. Available from: 10.1002/1097-0185(20010301)262:3<301::AID-AR1045>3.0.CO;2-H 11241198

[joa13869-bib-0062] Peters, S.E. & Rick, C. (1977) The actions of three hamstring muscles of the cat: a mechanical analysis. Journal of Morphology, 152(3), 315–327. Available from: 10.1002/jmor.1051520304 875040

[joa13869-bib-0063] Pincheira, P.A. , Boswell, M.A. , Franchi, M.V. , Delp, S.L. & Lichtwark, G.A. (2022) Biceps femoris long head sarcomere and fascicle length adaptations after 3 weeks of eccentric exercise training. Journal of Sport and Health Science, 11(1), 43–49. Available from: 10.1016/j.jshs.2021.09.002 34509714 PMC8847943

[joa13869-bib-0064] Purslow, P.P. (2020) The structure and role of intramuscular connective tissue in muscle function. Frontiers in Physiology, 11, 495. Available from: 10.3389/fphys.2020.00495 32508678 PMC7248366

[joa13869-bib-0065] Ranne, J.O. , Kainonen, T.U. , Lehtinen, J.T. , Kanto, K.J. , Vastamäki, H.A. , Kukkonen, M.K. et al. (2020) Arthroscopic coracoclavicular ligament reconstruction of chronic acromioclavicular dislocations using autogenous semitendinosus graft: a two‐year follow‐up study of 58 patients. Arthroscopy, Sports Medicine, and Rehabilitation, 2(1), e7–e15. Available from: 10.1016/j.asmr.2019.10.003 32266353 PMC7120851

[joa13869-bib-0066] Richmond, F.J.R. & Abrahams, V.C. (1975a) Morphology and distribution of muscle spindles in dorsal muscles of the cat neck. Journal of Neurophysiology, 38(6), 1322–1339. Available from: 10.1152/jn.1975.38.6.1322 1221076

[joa13869-bib-0067] Richmond, F.J.R. & Abrahams, V.C. (1975b) Morphology and enzyme histochemistry of dorsal muscles of the cat neck. Journal of Neurophysiology, 38(6), 1312–1321. Available from: 10.1152/jn.1975.38.6.1312 176329

[joa13869-bib-0068] Roy, R.R. , Powell, P.L. , Kanim, P. & Simpson, D.R. (1984) Architectural and histochemical analysis of the semitendinousus muscle in mice, rats, Guinea pigs, and rabbits. Journal of Morphology, 181(2), 155–160. Available from: 10.1002/jmor.1051810204 6481808

[joa13869-bib-0069] Sasahara, J. , Takao, M. , Miyamoto, W. , Oguro, K. & Matsushita, T. (2014) Partial harvesting technique in anterior cruciate ligament reconstruction with autologous semitendinosus tendon to prevent a postoperative decrease in deep knee flexion torque. The Knee, 21(5), 936–943. Available from: 10.1016/j.knee.2014.04.011 25017483

[joa13869-bib-0070] Sharafi, B. & Blemker, S.S. (2011) A mathematical model of force transmission from intrafascicularly terminating muscle fibers. Journal of Biomechanics, 44(11), 2031–2039. Available from: 10.1016/j.jbiomech.2011.04.038 21676398 PMC3134549

[joa13869-bib-0071] Smilde, H.A. , Vincent, J.A. , Baan, G.C. , Nardelli, P. , Lodder, J.C. , Mansvelder, H.D. et al. (2016) Changes in muscle spindle firing in response to length changes of neighboring muscles. Journal of Neurophysiology, 115(6), 3146–3155. Available from: 10.1152/jn.00937.2015 27075540 PMC4946610

[joa13869-bib-0072] Snow, B.J. , Wilcox, J.J. , Burks, R.T. & Greis, P.E. (2012) Evaluation of muscle size and fatty infiltration with MRI nine to eleven years following hamstring harvest for ACL reconstruction. Journal of Bone and Joint Surgery, 94(14), 1274–1282. Available from: 10.2106/JBJS.K.00692 22810397

[joa13869-bib-0073] Speedtsberg, M.B. , Zebis, M.K. , Lauridsen, H.B. , Magnussen, E. & Hölmich, P. (2022) Anatomical retraction of the semitendinosus muscle following harvest of the distal semitendinosus tendon for ACL reconstruction. Knee Surgery, Sports Traumatology, Arthroscopy, 30(5), 1706–1710. Available from: 10.1007/s00167-021-06718-1 34471958

[joa13869-bib-0074] Stenroos, A.J. & Brinck, T. (2020) Achilles tendon reconstruction with semitendinous tendon grafts is associated with a high complication rate. Journal of the American Podiatric Medical Association, 110(2), 3. Available from: 10.7547/18-014 32556230

[joa13869-bib-0075] Street, S.F. (1983) Lateral transmission of tension in frog myofibers: a myofibrillar network and transverse cytoskeletal connections are possible transmitters. Journal of Cellular Physiology, 114(3), 346–364. Available from: 10.1002/jcp.1041140314 6601109

[joa13869-bib-0076] Takahashi, K. , Kamibayashi, K. & Wakahara, T. (2021) Muscle size of individual hip extensors in sprint runners: its relation to spatiotemporal variables and sprint velocity during maximal velocity sprinting. PLoS One, 16(4), e0249670. Available from: 10.1371/journal.pone.0249670 33819316 PMC8021153

[joa13869-bib-0077] Tardieu, C. , Demirhan, O. , Akbal, E. , Ozgozen, L. , Biçer, Ö.S. , Delapré, A. et al. (2022) Modifications of the locomotor system in habitually quadrupedal humans. Journal of Anatomy, 241(3), 765–775. Available from: 10.1111/joa.13693 35661351 PMC9358744

[joa13869-bib-0078] Thaunat, M. , Fayard, J.M. & Sonnery‐Cottet, B. (2019) Hamstring tendons or bone‐patellar tendon‐bone graft for anterior cruciate ligament reconstruction? Orthopaedics and Traumatology: Surgery and Research, 105(1), S89–S94. Available from: 10.1016/j.otsr.2018.05.014 30130660

[joa13869-bib-0079] Trotter, J.A. , Richmond, F.J.R. & Purslow, P.P. (1995) Functional morphology and motor control of series‐fibered muscles. Exercise and Sport Sciences Reviews, 23(1), 167–213. Available from: 10.1249/00003677-199500230-00008 7556350

[joa13869-bib-0080] van der Made, A.D. , Wieldraaijer, T. , Kerkhoffs, G.M. , Kleipool, R.P. , Engebretsen, L. , van Dijk, C.N. et al. (2015) The hamstring muscle complex. Knee Surgery, Sports Traumatology, Arthroscopy, 23(7), 2115–2122. Available from: 10.1007/s00167-013-2744-0 24190369

[joa13869-bib-0081] Van Dyke, J.M. , Bain, J.L.W. & Riley, D.A. (2012) Preserving sarcomere number after tenotomy requires stretch and contraction. Muscle & Nerve, 45(3), 367–375. Available from: 10.1002/mus.22286 22334171

[joa13869-bib-0082] Vertullo, C.J. , Piepenbrink, M. , Smith, P.A. , Wilson, A.J. & Wijdicks, C.A. (2019) Biomechanical testing of three alternative quadrupled tendon graft constructs with adjustable loop suspensory fixation for anterior cruciate ligament reconstruction compared with four‐strand grafts fixed with screws and femoral fixed loop devices. American Journal of Sports Medicine, 47(4), 828–836. Available from: 10.1177/0363546518825256 30789779

[joa13869-bib-0083] Virtanen, K.J. , Savolainen, V. , Tulikoura, I. , Remes, V. , Haapamäki, V. , Pajarinen, J. et al. (2014) Surgical treatment of chronic acromioclavicular joint dislocation with autogenous tendon grafts. Springerplus, 3, 420. Available from: 10.1186/2193-1801-3-420 25152850 PMC4141074

[joa13869-bib-0084] Wakahara, T. , Ema, R. , Miyamoto, N. & Kawakami, Y. (2015) Increase in vastus lateralis aponeurosis width induced by resistance training: implications for a hypertrophic model of pennate muscle. European Journal of Applied Physiology, 115(2), 309–316. Available from: 10.1007/s00421-014-3012-9 25294665

[joa13869-bib-0085] Wickiewicz, T.L. , Roy, R.R. , Powell, P.L. & Edgerton, V.R. (1983) Muscle architecture of the human lower limb. Clinical Orthopaedics and Related Research, 179, 275–283. Available from: 10.1097/00003086-198310000-00042 6617027

[joa13869-bib-0086] Wickiewicz, T.L. , Roy, R.R. , Powell, P.L. , Perrine, J.J. & Edgerton, V.R. (1984) Muscle architecture and force‐velocity relationships in humans. Journal of Applied Physiology: Respiratory, Environmental, and Exercise Physiology, 57(2), 435–443. Available from: 10.1152/jappl.1984.57.2.435 6469814

[joa13869-bib-0087] Williams, G.N. , Snyder‐Mackler, L. , Barrance, P.J. , Axe, M.J. & Buchanan, T.S. (2004) Muscle and tendon morphology after reconstruction of the anterior cruciate ligament with autologous semitendinosus‐gracilis graft. Journal of Bone and Joint Surgery, 86(9), 1936–1946. Available from: 10.2106/00004623-200409000-00012 15342756

[joa13869-bib-0088] Wisdom, K.M. , Delp, S.L. & Kuhl, E. (2015) Use it or lose it: multiscale skeletal muscle adaptation to mechanical stimuli. Biomechanics and Modeling in Mechanobiology, 14(2), 195–215. Available from: 10.1007/s10237-014-0607-3 25199941 PMC4352121

[joa13869-bib-0089] Woodley, S.J. , Duxson, M.J. & Mercer, S.R. (2007) Preliminary observations on the microarchitecture of the human abdominal muscles. Clinical Anatomy, 20(7), 808–813. Available from: 10.1002/ca.20523 17708566

[joa13869-bib-0090] Woodley, S.J. & Mercer, S.R. (2005) Hamstring muscles: architecture and innervation. Cells, Tissues, Organs, 179(3), 125–141. Available from: 10.1159/000085004 15947463

[joa13869-bib-0091] Zhang, C. & Gao, Y. (2012) Finite element analysis of mechanics of lateral transmission of force in single muscle fiber. Journal of Biomechanics, 45(11), 2001–2006. Available from: 10.1016/j.jbiomech.2012.04.026 22682257 PMC3843153

